# An unusual presentation of a stroke in a developing country: a case report

**DOI:** 10.1186/s13104-017-2378-2

**Published:** 2017-01-26

**Authors:** N. D. B. Ehelepola, T. I. D. M. Ranasinghe, B. Prashanthi, H. M. P. A. G. S. Bandara

**Affiliations:** The Teaching (General) Hospital-Kandy, Kandy, Sri Lanka

**Keywords:** Cerebrovascular accident, Cerebral hemorrhage, Accidental falls, Sri Lanka, Brain-computed tomography, Occupational injuries, Case report

## Abstract

**Background:**

Patients often, but not always, present with features that allow easy differentiation between traumatic brain injury and a stroke. Early diagnosis and appropriate treatment are crucial for a good outcome in both. Millions of people worldwide climb coconut and other trees without any protective gear. We present a case of a coconut tree climber found unconscious after a fall, initially misdiagnosed as a traumatic brain injury but later proven to be a hemorrhagic stroke. We discuss how to prevent such incidents and why that deserves more attention. There is a severe paucity of such case reports and discussion of related issues in medical literature.

**Case presentation:**

A 65 year old, previously healthy Sinhalese man had fallen from a coconut tree and was found unconscious with wounds on his limbs on the right side of his body. He was taken to the closest hospital. After being given primary care, he was transferred to the Kandy teaching hospital for neurosurgical management. Physical examination findings suggested a stroke but not the medical history. We could not exclude a head and cervical spine injury clinically. A computed tomography scan of the brain and cervical spine showed a left thalamic hemorrhage but no other injuries that could be attributed to trauma, therefore we confirmed it was a hemorrhagic stroke presenting as a traumatic brain injury.

**Conclusion:**

Since strokes are very common and rising in prevalence, being more aware of uncommon presentations like this can be useful to all health care workers working in acute medical settings especially in developing countries. Computed tomography scans of the brain plays a critical role in accurate diagnosis of both strokes and traumatic brain injuries; but many people in the developing world do not have prompt access to computed tomography scanners. Providing early access to a computed tomography scan of the brain to a wider population after a head injury or a stroke may contribute to reducing morbidity and mortality. Developing and promoting affordable and simple safety methods for palm and other tree climbers can also help to reduce morbidity and mortality.

## Background

Strokes and head injuries are common medical and surgical causes for emergency hospital admission worldwide and these are managed differently at different departments of a larger hospital with computed tomography (CT) scanners. Many smaller hospitals do not have the facilities to optimally manage both of them. Early diagnosis and direction to the appropriate hospital for treatment is important for a better outcome in both conditions. Reported cases of both head injuries and strokes are on the rise in Sri Lanka and worldwide. Patients usually present with histories which are easy to differentiate between a traumatic brain injury and a stroke but not always as illustrated by this case.

Annually, 15 million people suffer a stroke worldwide [1] and elderly people are mostly affected. Since strokes are very common, being conscious of uncommon presentations like this will be useful to all health care workers working in acute medical settings, especially in developing countries.

## Case presentation

A 65-year old Sinhalese coconut-tree-climber fell four meters to the ground one morning in the Kandy district, Sri Lanka. He was found unconscious and unconsciousness lasted about 10 min. There were lacerations on his right elbow, below his right knee and abrasions on his chin and the right of his face. Movements of the limbs on his right were reduced.

He was taken to the closest government hospital by an auto rickshaw (without any support to the spine).There, he was examined, basic life support was given, the wounds were dressed and he was then transferred to the Kandy teaching hospital (T.H. Kandy), the only hospital with a neurosurgical unit plus a CT scanner in the province, by ambulance.

Eight and half hours after the fall, he arrived at the T.H. Kandy. On arrival his pulse rate was 92/min, his blood pressure was 120/80 mm mercury, his respiratory rate was 28/min, his lungs and abdomen were normal on examination. His level of consciousness was 15/15 in Glasgow coma scale and there was slurring of speech, pupils were equal central and were reacting to light. His sphincter functions were intact.

He had a headache and there was some tenderness on the right of his scalp but there were no scalp wounds. He had had no past history of attacks of impaired consciousness, seizures or other neurological problems, hypertension, diabetes or any cardiac diseases; He was, however, a smoker.

**Fig. 1:**
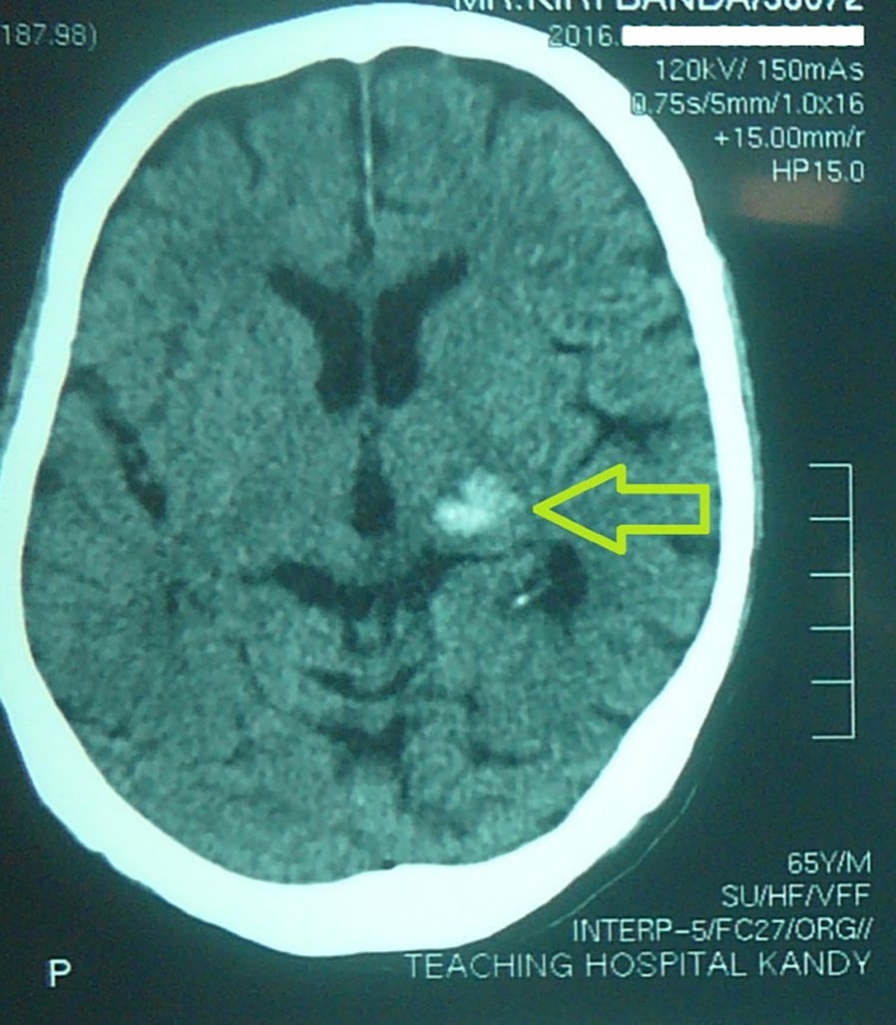
Computed tomography scan of the brain of the patient. The *arrowhead* pointed at hemorrhages in the left thalamic area

This patient could not recall if he had developed a headache or any weakness prior to his fall. X-rays of his limbs on the right and his chest did not show any fractures but a non contrast computed tomography scan (NCCT) of the head and cervical spine showed an intracerebral hemorrhage (ICH) in the left thalamic area suggestive of a hemorrhagic stroke without any evidence of a traumatic injury as shown in Fig. 1.

A more detailed neurological examination was carried out. Increased muscle tone and reduced muscle power, exaggerated tendon reflexes and the Babinski sign on the right were found. This deep seated ICH was small and his blood pressure was not high, hence the risk of enlarging of ICH was low. There are differences in the way of medical management of hemorrhagic strokes among doctors we know. After getting the opinion of neurosurgical team we came to a consensus about the way to manage this small and deep seated ICH medically. 20% mannitol 250 ml was infused twice daily for two days and phenytoin sodium 100 mg twice daily was started as prophylactic drugs.

The wounds were cleaned and dressed. A course of physiotherapy was arranged. As there was shortage of beds in our unit, the patient was re-transferred to the local hospital by ambulance for continuation of management.

After three days he was able to move the limbs on his right and was sent home and asked to come regularly to get his wounds cleaned and dressed. No medication was prescribed at discharge. It took six weeks for his right leg wound to completely heal and there was progressive improvement of muscle power of the limbs on his right but that progress stopped after two months. After three months he was able to walk with support or with a walking stick.

If a previously healthy person comes to a hospital having fallen from a height and becoming unconscious without being able to move their limbs after regaining consciousness, the first differential diagnosis that comes to a doctor’s mind most is a head injury (traumatic brain injury) with a cervical spine injury. In this case, the patient had wounds on both limbs on his right and that may also have been a reason not to move them.

As stated earlier, the final diagnosis was a hemorrhagic stroke. Nevertheless we cannot be 100% certain of that diagnosis as there are few reported cases of similar thalamic/basal ganglia bleeds following trauma [2]. But what makes this case remarkable was the stroke happened while climbing a tree making a quick diagnosis more complex.

The latest guidelines for the management of spontaneous intracerebral hemorrhages by the American heart association and American stroke association says that mannitol may be used to treat acute intracranial pressure elevations (not prophylactically) and does not recommend prophylactic use of antiseizure medication like phenytoin [3]. However, the latest European stroke organization guidelines say that there is insufficient evidence to make strong recommendations on whether prophylactic antiepileptic treatment should be used after ICH [4]. Mannitol is being used routinely in some other countries as well and some recent studies show benefit of mannitol in patients with spontaneous ICH [5].

Rather than focusing only on this patient, we would like to discuss some wider issues, pertaining to diagnosis and prevention of similar incidents taking this case as an example as this can be useful to society. Prevention of strokes is very important to prevent incidents like this case. We are not, however, going to discuss stroke prevention in this paper since that topic receives deserving attention in medical literature.

**Fig. 2:**
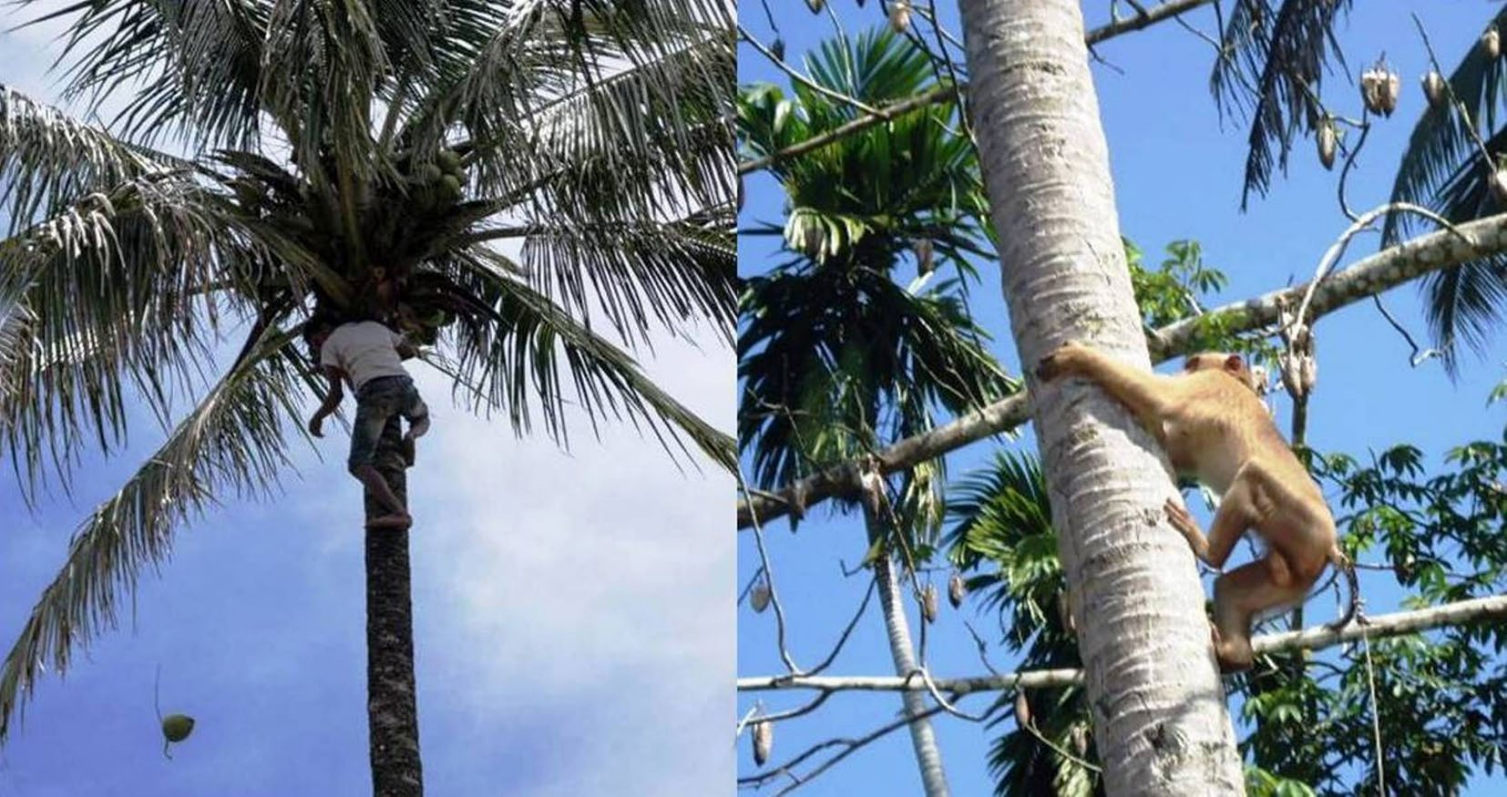
A Timorese man harvesting coconuts [7] in the same way as the majority of other people around the world still do. A trained monkey climbing a coconut tree in Malaysia to harvest coconut (in the *right panel*) for comparison. There is a need and room for improvement of the safety of technique used by humans for this job

Coconuts and other palm trees are very important tropical crops. Palm trees are depicted in national emblems of world’s nations more than any other trees, which is testament to their importance. In some 90 countries, more than 11 million farmers depend on coconuts for their livelihoods [6]. Thousands of people like this patient climb coconut trees daily to earn a living in Sri Lanka alone. The great majority do that without any protective gear as depicted in the Fig. 2.

Sri Lanka annually produced 2.5–3.0 billion coconuts in 2006–2015 [8] and most were harvested this way. This occupation is unpopular in younger generations in Sri Lanka which makes older people continue to work. Palm trees usually grow vertical without branches. Hence, in a case of the climber feeling faintish, or slipping his grip on trunk, nothing is there to support his body.

In Sri Lanka and many other countries population aged over 60 is rising and retiring age is also rising [9]. Age is the single most important risk factor for stroke [10]. This links with the fact that tree climbers in Sri Lanka tend to be of the older generation due to it being unpopular in younger people. There were some past efforts to improve the safety of tree climbers [11] but those did not gain wide acceptance among them. Additional to tree climbers, there are other industries where workers are at risk of falls from heights.

Often, there is no time to perform a complete neurological examination on the initial examination of a patient presented with a similar history. This is especially true at Sri Lankan government hospitals that provide services free of charge. Congestion is a further issue in this regard. Usually the doctors try to identify the greatest threat/s to life and treat them and then do a detailed examination [12]. First we look for treatable traumatic brain injury and cervical spine injury in such a patient, and only after exclusion of these, do we consider rarer possibilities.

This case highlights the crucial role of an early CT scan of the brain and cervical spine in order to obtain an early, detailed and accurate diagnosis of such patients. Furthermore, when a stroke has been clinically diagnosed, an early NCCT of the brain is essential to differentiate between a stroke that has been caused by an infarct and one that has occurred due to a hemorrhage. This detection is crucial since the treatment differs. However, CT scanners are expensive and only a few major hospitals in Sri Lanka and other developing countries have them and it is important to do a detailed neurological examination in such patients early.

Early identification of infarcts and thrombolytic therapy with recombinant tissue plasminogen activator within three hours of onset improves the outcome in most ischemic strokes [13], and most strokes in fact are due to ischemia. Education of the public and the staff of smaller healthcare institutions of the importance of taking the patient within 3 h directly to a hospital with facilities in a case a person develops symptoms of stroke is important (going via other institutions costs time). However, that may aggravate existing overcrowding of big hospitals. This patient could have been taken to T.H. Kandy in less than an hour but the on lookers (not the family) hired an auto rickshaw and took him to the closest hospital probably because it was the easiest and cheapest way to help him.

Had the CT scan of this patient shown a larger hemorrhage with evidence of increased intracranial pressure with a fracture of the skull, if the hemorrhage was due to a stroke and fracture was due to the fall, the diagnosis would have been more difficult.

Had this patient got a transient ischemic attack (TIA), and had the fall and then got a fracture of skull with a large ICH, again the chances for missing detection of TIA are very high.

37.3 million falls that are severe enough to require medical attention, occur each year worldwide [14]. Falls from heights are the second leading cause of unintentional injury deaths worldwide of which over 80% are in low- and middle-income countries [15] and mostly elderly people are affected. Of all types of injury, those to the brain are among the most likely to result in death or permanent disability [16]. Globally the numbers of reported cases of strokes are also on the increase [1], with 87% of strokes occurring in low- and middle-income countries [16]. Asia is the most populated continent and home to the largest number of tree climbers. The overall incidence of strokes in Asia is between 116 and 483/100,000 per year [17]. If we consider the Sri Lankan situation, according to the 2013 annual health bulletin of the Sri Lanka health ministry, cerebro vascular diseases was the third leading cause of hospital deaths in 2013, it was the fifth leading cause in 2005, the fourth leading cause from 2006 to 2012 [18]. According to the World Health Organization strokes were the second leading causes of death in Sri Lanka, being responsible for 15.2% of all deaths in 2012 [19]. Years of life lost (YLL) due to strokes in Sri Lanka in 2010 was estimated to be 209,000 [20]. Injuries were the leading cause of hospital admissions in Sri Lanka for the past two decades [21]. According to a recent study done in Kandy district the commonest mode of injury was falling from heights [22].

Considering everything, we were surprised to find the paucity of similar cases being reported and the lack of discussion about the related issues in earlier medical literature. We expect more cases in the coming years similar to this patient, especially in the developing world. Considering the enormous burden in managing them, giving more attention to primary prevention is important.

There are districts in Sri Lanka and in other developing countries where there is no hospital with a scanner for brain imaging. The above facts illustrate the importance of have better access to CT scanners within a reasonable time after a possible stroke or a head injury, to a wider population. The opening of dedicated stroke units at large hospitals may become a necessity in Sri Lanka (and other developing countries) in the near future because of the rising expectations of the people.

An increase in the risk of hemorrhagic strokes after traumatic brain injuries was demonstrated epidemiologically [23] but they are considered risky only after some weeks and this patient’s brain CT showed a typical hemorrhagic stroke with no evidence to suggest a traumatic brain injury.

## Conclusion

It is important to consider a stroke as a possibility when a patient aged over 60 years presents with loss of consciousness and hemiparesis after a fall from a height. Education of the public to take such patients to a larger hospital (with a CT scanner and facilities for thrombolysis and neurosurgery) as early as possible using appropriate transportation methods (the spine stabilized) may help to reduce morbidity and mortality. Considering the rising and already huge, aging population who climb coconut palm and other trees daily, the introduction and promotion of safety measures to minimize falls is important.

## Abbreviations

CT: computed tomography
T.H. Kandy: Kandy Teaching Hospital
NCCT: non contrast computed tomography scan
ICH: intracerebral hemorrhage
TIA: transient ischemic attack
YLL: years of life lost
